# Neurofibromatosis type 1 with subarachnoid hemorrhage due to multiple and *de novo* aneurysms: a case report

**DOI:** 10.1186/s13256-021-02967-3

**Published:** 2021-07-30

**Authors:** Tatsuya Uchida, Kenichi Amagasaki, Atsushi Hosono, Hiroshi Nakaguchi

**Affiliations:** Department of Neurosurgery, Mitsui Memorial Hospital, 1 Kandaizumicho, Chiyoda-ku, Tokyo, 101-8643 Japan

**Keywords:** Neurofibromatosis type 1, Subarachnoid hemorrhage, *De novo* aneurysms, Case report

## Abstract

**Background:**

Neurofibromatosis type 1 causes various lesions in many organs including the skin, and the incidence of complications with intracranial aneurysms is 9–11%. Here we report a case of neurofibromatosis type 1 with subarachnoid hemorrhage due to multiple and *de novo* aneurysms.

**Case presentation:**

The patient was a 49-year-old Japanese woman with a history of neurofibromatosis type 1. She was transported to our hospital owing to disturbance of consciousness and was diagnosed with subarachnoid hemorrhage by computed tomography. Computed tomography angiography revealed multiple, small intracranial aneurysms, and we suspected that one of them in the peripheral branch of the left middle cerebral artery was the source of hemorrhage based on the distribution of hematoma. The patient underwent emergency surgery. Because it was difficult to identify an aneurysm in the most peripheral part of the left middle cerebral artery in the initial surgery, only one aneurysm was clipped. Later, a peripheral aneurysm was clipped using the navigation system. Because both aneurysms were small intracranial aneurysms (< 2 mm), either of them could be the source of hemorrhage. The postoperative course was good, and the patient was discharged in healthy condition. Because brain magnetic resonance imaging performed in the previous year did not find aneurysms at the same site, she was diagnosed with rupture of a *de novo* aneurysm. Neurofibromatosis type 1 might have caused the rupture of multiple intracranial aneurysms in a short period in this patient.

**Conclusion:**

Neurofibromatosis type 1 may be complicated by the formation of multiple intracranial aneurysms in a short period.

## Introduction

Neurofibromatosis type 1 (NF1) causes various lesions in many organs, including the skin [1]. The estimated number of patients with NF1 is about 40,000 in Japan, and the prevalence is about 1 in of every 3000 people. There is no racial difference in the morbidity [2]. It is an autosomal dominant genetic disease, and more than half of the patients have sporadic NF1, which is caused by a mutation of a gene on the long arm of chromosome 17q11.2. that is responsible for production of neurofibromin [2, 3]. Well-known complications in the central nervous system associated with this disease include neurofibroma and optic glioma. In addition, some studies have demonstrated cardiovascular anomalies in NF1, such as moyamoya disease, internal carotid artery occlusion or stenosis, cerebral arteriovenous fistula, dissection, or intracranial aneurysms [4–13]. Here, we report a case of NF1 with subarachnoid hemorrhage due to multiple and *de novo* aneurysms.

## Case presentation

**Fig. 1.**
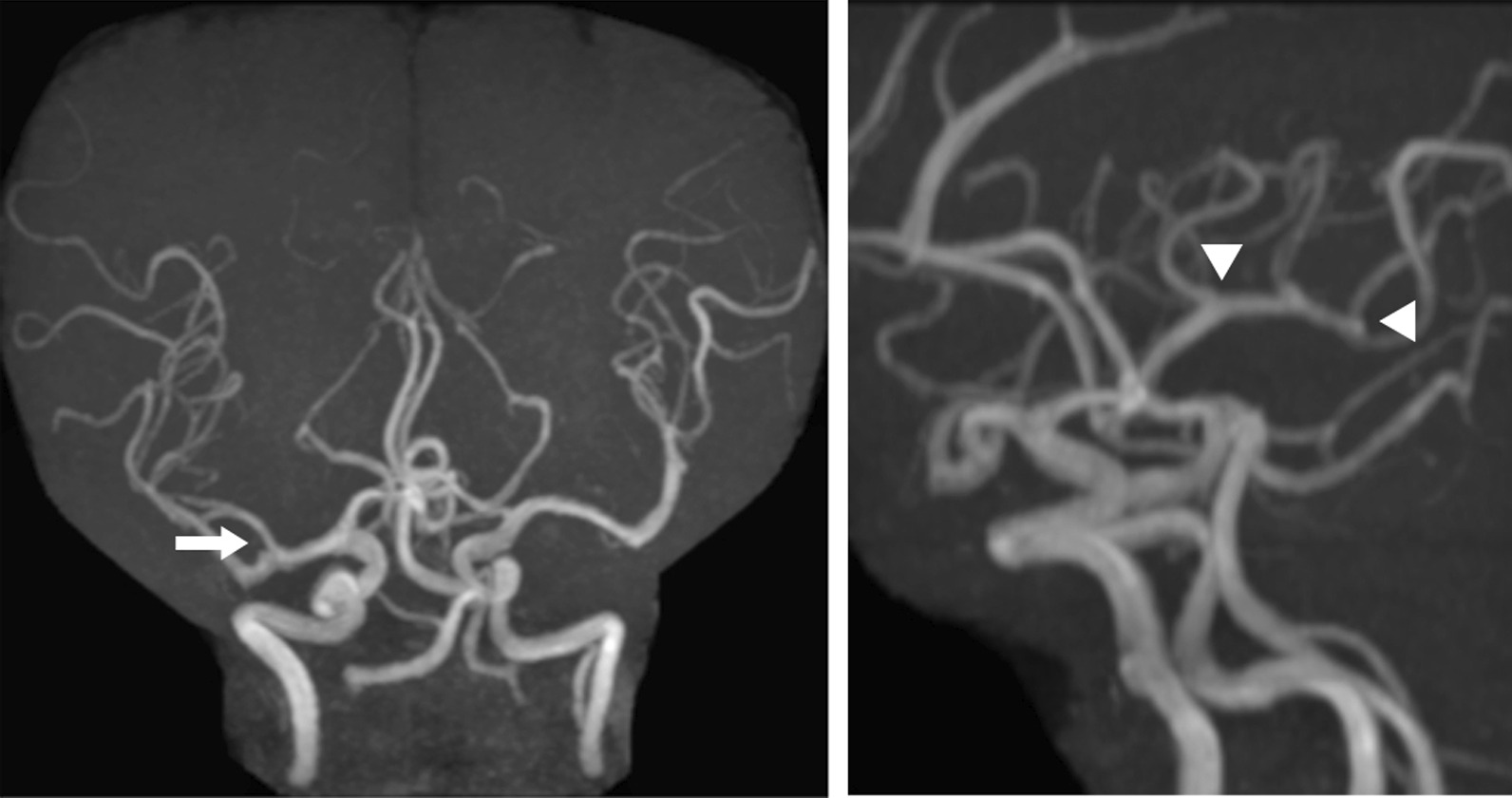
Magnetic resonance imaging (MRI) in the previous year. Left: because a small intracranial aneurysm (arrow) was found in the right middle cerebral artery 2 years ago, she underwent annual MRI follow-up. Right: in the previous year, there had been no aneurysms in the left middle cerebral artery where the aneurysms were (arrow head)

The patient was a 49-year-old Japanese woman with a history of NF1. There was no family history. She had brown spots on the skin and multiple dermatofibromas, and she was diagnosed with sporadic NF1 by biopsy in her teens. After small intracranial aneurysms (< 2 mm) were found in the right middle cerebral artery 2 years ago, she underwent annual magnetic resonance imaging (MRI) follow-up (Fig. 1 left). In addition, she was orally administered phenytoin for the treatment of genuine epilepsy. There were no aneurysms anywhere other than in the brain. She also had a history of untreated hypertension. She had no other complications, such as intracranial neurofibroma and brain tumors.

**Fig. 2.**
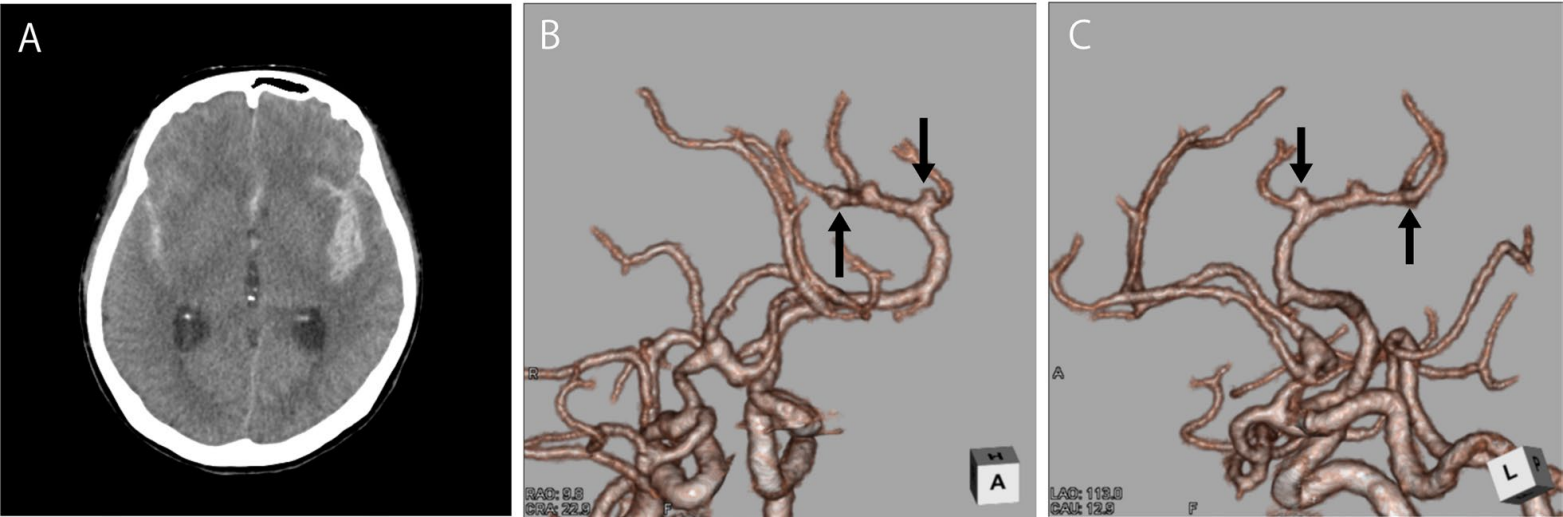
Subarachnoid hemorrhage due to the rupture of intracranial aneurysms. The patient was transported to our hospital because of acute headache and disturbance of consciousness. **A** Computed tomography (CT) showing subarachnoid hemorrhage. **B **(anterior view),** C** (lateral view), CT angiography revealing multiple intracranial aneurysms (< 2 mm) (arrow). Because brain magnetic resonance imaging performed in the previous year did not find aneurysms at the same site, she was diagnosed with rupture of a *de novo* aneurysm

**Fig. 3.**
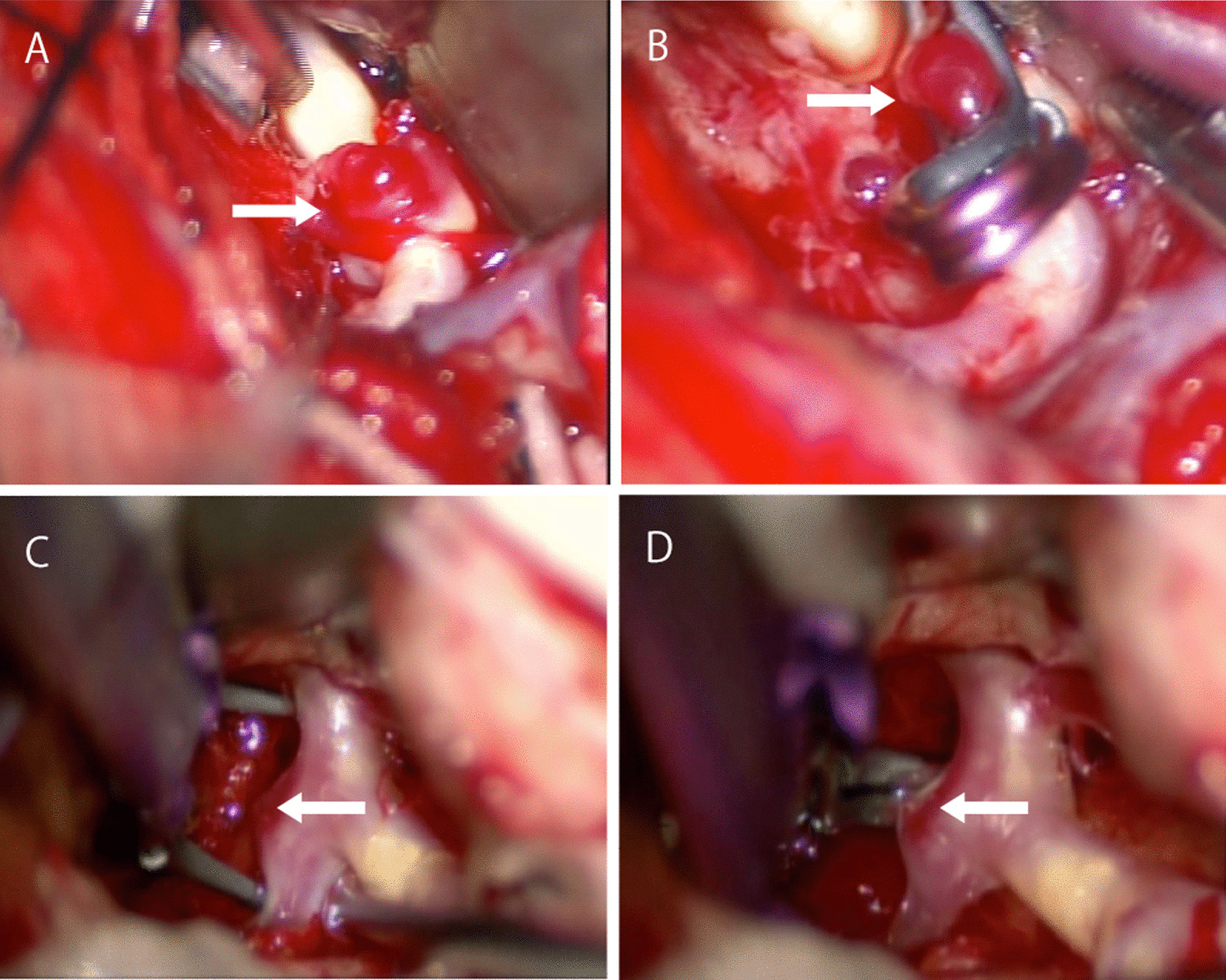
Intraoperative photographs. In the initial surgery, only one small aneurysm was clipped because it was difficult to identify another one, which was in the most peripheral part of the left middle cerebral artery. **A** The aneurysm (arrow) in the bifurcation of the left middle cerebral artery was exposed. **B** Clipping was performed. In the second surgery, the peripheral aneurysm was identified using the Navigation System. **C** The aneurysm (arrow) was exposed. **D** Clipping was performed

**Fig. 4.**
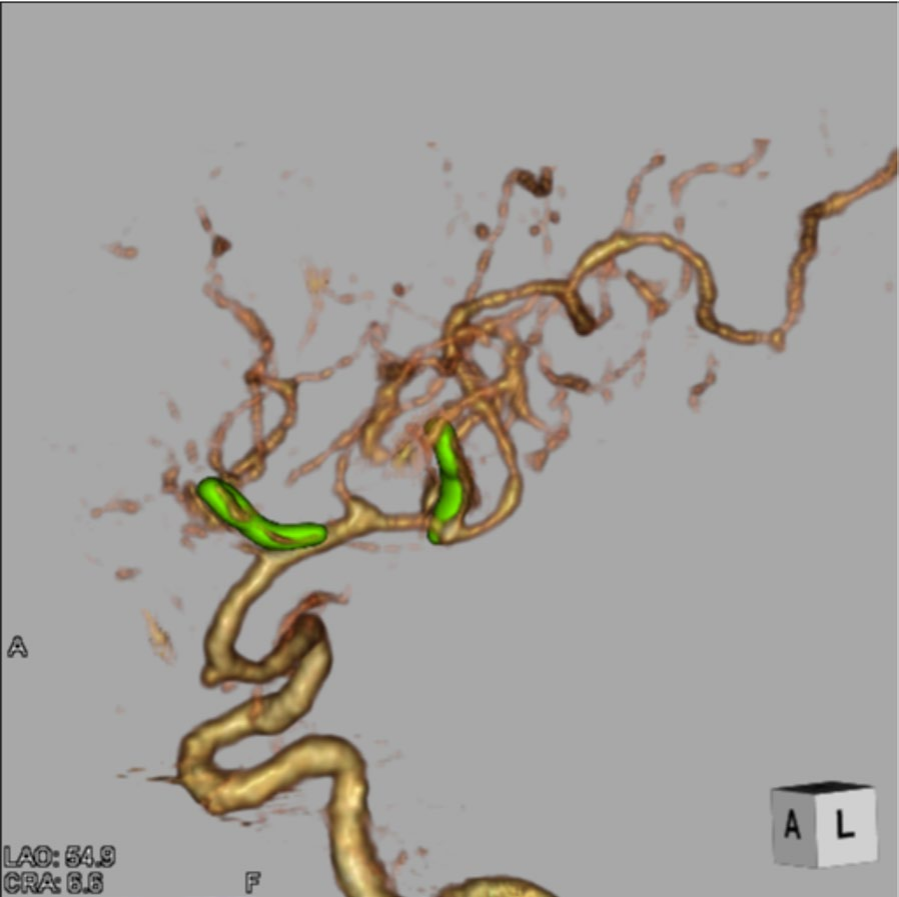
Postoperative images. The aneurysms had disappeared on computed tomography angiography after the operations

She was transported to our hospital because of acute headache and disturbance of consciousness and was diagnosed with subarachnoid hemorrhage by computed tomography (CT) (Fig. 2A). Regarding the disturbance of consciousness, she had a score of 13 on the Glasgow Coma Scale, grade III in the Hunt and Kosnik grading system, and grade II in the World Federation of Neurosurgical Societies grading. Blood pressure was over 200 mmHg. Computed tomography angiography revealed multiple intracranial aneurysms (Fig. 2B, and C). Based on the distribution of hematoma, we suspected that one of the aneurysms in the peripheral branch of the left middle cerebral artery, rather than known intracranial aneurysms in the right middle cerebral artery, was the source of hemorrhage. These multiple small intracranial aneurysms in the left middle cerebral artery were considered *de novo* aneurysms because they were not found on brain MRI in the previous year (Fig. 1 right). Accordingly, she underwent emergency surgery on the day of admission. After a left frontotemporal craniotomy, the Sylvian fissure was opened, exposing the superior and inferior trunk (M2) of the middle cerebral artery. We found small intracranial aneurysm (< 2 mm) in the bifurcation of the left middle cerebral artery. During surgery, only one small aneurysm was clipped (Fig. 3A, and B) because it was difficult to identify another one, which was in the most peripheral part of the left middle cerebral artery. Three days later, the peripheral aneurysm, which was difficult to be identified in the initial surgery, was clipped and wrapped using the Navigation System (Stealth Station S7 Surgical Navigation System, Medtronic Inc., Minneapolis, Minnesota, USA) (Fig. 3C, and D). The small distal intracranial aneurysm, which was clipped during the second surgery, was considered the source of rupture because hematoma was around the aneurysm, and the wall was slightly red. However, because both aneurysms were small intracranial aneurysms (< 2 mm), either of them could be the source of hemorrhage. We confirmed that the aneurysms had disappeared by computed tomography angiography after the operations (Fig. 4). The postoperative course was good without any complications, such as rebleeding, cerebrovascular spasm, and hydrocephalus, and she was discharged in a healthy condition. The modified Rankin Scale score at discharge was 1. She continued to undergo follow-up MRIs of the known right middle cerebral artery aneurysm.

## Discussion

Neurofibromatoses encountered in the field of neurosurgery are mostly neoplastic diseases, such as acoustic neuroma, meningioma, and optic glioma. While moyamoya vessels [4–6], internal carotid artery occlusion, and stenosis [9, 11] are common cerebrovascular lesions, dural arteriovenous fistula [5, 14] and cerebral artery dissection [8] have also been identified in NF1 patients. To date, not many case reports have investigated complications of intracranial aneurysms [10, 12, 13, 15, 16]. Although the incidence of intracranial aneurysms is higher in NF1 patients (9–11%) than in age-matched individuals [17, 18], screening for intracranial aneurysms is considered unnecessary [18]. Fukunaga *et al*. found that, in NF1 patients, the internal carotid artery was the most common (32%) site of aneurysm formation, and the incidence of multiple aneurysms was 32%, which was slightly higher than that of typical intracranial aneurysms [19].

There are many arguments about factors contributing to the formation of intracranial aneurysms in NF1 patients, and no consensus has been reached on this issue. Hemodynamic factors associated with the carotid artery occlusion, stenosis, and growth of moyamoya vessels may be responsible for the formation of intracranial aneurysms in NF1 patients [11]. However, vascular anomalies, such as carotid artery occlusion and collateral blood flow, were not observed in the patient in this study, suggesting that hemodynamic factors are not involved in this case.

Salyer *et al*. performed the histopathological examination and speculated the mechanism underlying renal artery lesions as follows: (1) Schwann cells proliferate in the vascular wall, and (2) the vascular wall then becomes fragile owing to fibrous intimal thickening, defects of the medial smooth muscle, and destruction of the elastic lamina [20]. However, it has been pointed out that the mechanisms underlying the formation of aneurysms in the brain vessels cannot be same as those in renal vessels because nerves are unmyelinated, and Schwann cells never proliferate in the central blood vessels [21]. Sobata *et al*. found that degeneration and loss of smooth muscle cells triggered by the destruction of the internal elastic lamina led to the formation of aneurysms in intracranial vessels [21].

NF is often complicated by hypertension. In this case, the patient also had a history of untreated hypertension [20]. Gibbons *et al*. have demonstrated an association between pheochromocytoma-associated hypertension and subarachnoid hemorrhage due to the rupture of intracranial aneurysms [22]. In addition, Hasegawa *et al*. reported a case of multiple intracranial aneurysms complicated by hypertension and catecholamine-secreting malignant schwannoma, and their findings indicate that hypertension is involved in the formation of multiple intracranial aneurysms [23].

As mentioned above, no consensus has been reached on the mechanism underlying the formation of intracranial aneurysms. The NF1 patient with vascular fragility in this study had hypertension, which might have been involved in the formation and rupture of *de novo* aneurysms in a short period.

Some studies suggest that screening for intracranial aneurysms is unnecessary in patients with NF1 [18], and other studies indicate no significant relationship between NF1 and the risk of subarachnoid hemorrhage [24]. However, the present patient was found to have a subarachnoid hemorrhage due to the rupture of *de novo* aneurysms, although she underwent annual brain MRI. Therefore, more frequent MRI monitoring of intracranial aneurysms is important in patients with NF1. In addition, further studies are needed to examine the mechanism underlying the formation of aneurysms using biopsies of aneurysms or the superficial temporal artery.

## Conclusion

Here, we report a case of NF1 with subarachnoid hemorrhage due to multiple and *de novo* aneurysms. NF1 may be complicated by the formation of multiple intracranial aneurysms in a short period.

## Data Availability

We make our references in the manuscript available for testing by reviewers.
